# Chemotherapeutic Agents Sensitize Resistant Cancer Cells to the DR5-Specific Variant DR5-B More Efficiently Than to TRAIL by Modulating the Surface Expression of Death and Decoy Receptors

**DOI:** 10.3390/cancers12051129

**Published:** 2020-04-30

**Authors:** Artem A. Artykov, Dmitry A. Belov, Victoria O. Shipunova, Daria B. Trushina, Sergey M. Deyev, Dmitry A. Dolgikh, Mikhail P. Kirpichnikov, Marine E. Gasparian

**Affiliations:** 1 Department of Bioengineering, Shemyakin-Ovchinnikov Institute of Bioorganic Chemistry, Russian Academy of Sciences, 117997 Moscow, Russia; art.al.artykov@gmail.com (A.A.A.); liege.highness@yandex.ru (D.A.B.); dolgikh@nmr.ru (D.A.D.); kirpichnikov@inbox.ru (M.P.K.); 2 Faculty of Biology, M.V. Lomonosov Moscow State University, 119992 Moscow, Russia; 3 Department of Immunology, Shemyakin-Ovchinnikov Institute of Bioorganic Chemistry, Russian Academy of Sciences, 117997 Moscow, Russia; vika_shipunova@mail.ru (V.O.S.); biomem@mail.ru (S.M.D.); 4 Department of X-ray and Synchrotron Research, A.V. Shubnikov Institute of Crystallography of Federal Scientific Research Centre “Crystallography and Photonics” of Russian Academy of Sciences, 119333 Moscow, Russia; trushina.d@mail.ru

**Keywords:** TRAIL, DR5-B, DR4, DR5, DcR1, DcR2, doxorubicin, bortezomib, panobinostat

## Abstract

TRAIL is considered a promising antitumor agent because it causes apoptosis of transformed cells without affecting normal cells. However, many types of tumors are cytokine resistant, and combination therapy with various chemotherapeutic drugs is being developed to overcome the resistance. We have demonstrated that the combination of TRAIL with doxorubicin, bortezomib, and panobinostat dramatically reduced the viability of TRAIL-resistant A549 and HT-29 cells. Chemotherapy even more efficiently sensitized cells to the DR5-specific mutant variant of TRAIL DR5-B, which does not have an affinity for decoy receptors. Bortezomib and doxorubicin greatly enhanced the surface expression of the death receptors DR5 and DR4, while panobinostat increased expression of DR5 and suppressed expression of DR4 in both cell lines. All drugs increased surface expression of the decoy receptors DcR1 and DcR2. Unlike the combined treatment, if the cells were pretreated with chemotherapy for 24 h, the cytotoxic activity of TRAIL was less pronounced, while sequential treatment of cells enhanced the effectiveness of DR5-B. The same results were obtained with agonistic anti-DR5 antibodies. Thus, the effectiveness of TRAIL was rather limited due to changes in the ratio of death and decoy receptors and DR5-specific agonists may be preferred in combination antitumor therapy regimens.

## 1. Introduction

The discovery that the cytokine TRAIL (tumor necrosis factor related apoptosis-inducing ligand) can cause apoptosis of cancer cells without causing the death of normal cells and without showing toxicity in mice and primates, has led to intensive studies on the mechanisms of TRAIL-induced apoptosis. It is known that TRAIL interacts with five receptors, four of which are expressed on the plasma membrane (DR4, DR5, DcR1, DcR2), and the fifth is a soluble osteoprotegerin receptor (OPG) [1]. Death receptors DR4 and DR5 contain the cytoplasmic death domain (DD), which is involved in the initiation of the apoptotic cascade. The binding of TRAIL to DR4 and DR5 causes the association of the FADD (Fas associated protein with death domain) adapter protein and procaspase-8 with the DD domains of death receptors forming DISC (death-inducing signaling complex), followed by activation of caspase-8, which leads to the launch of the apoptotic cascade [2,3]. The other three receptors do not transmit apoptosis signals and can inhibit TRAIL-mediated apoptosis. Decoy receptors DcR1 and DcR2 inhibit TRAIL-mediated apoptosis, preventing the assembly of DISC (death inducing signaling complex) by TRAIL titration or by recruiting DR5 to DISC [4,5]. Moreover, it was shown that receptors DcR1 and DcR2 not only act in a cell-autonomous or cis-regulatory manner, but also exert trans-cellular regulation [6].

Many cancer cells are resistant to TRAIL despite expression of death receptors on the cell surface [7]. The ratio of surface expression of death and decoy receptors can serve as a predictor of the cancer cells sensitivity to TRAIL. In addition, intracellular traffic and subcellular localization of death and decoy receptors are essential for TRAIL sensitivity [8,9]. Accumulation of death receptors in autophagosomes and the nucleus correlated with resistance of cells to TRAIL [10]. Impaired expression of death receptors on the surface of the plasma membrane is one of the main mechanisms for ensuring cell resistance to TRAIL [9].

To overcome the resistance of tumors to TRAIL, schemes of combination therapy regimens with known chemotherapeutic drugs and death receptor agonists have been used in clinical trials [11]. Many synthetic and natural compounds are able to restore the sensitivity of cells to TRAIL-dependent apoptosis by upregulation of death receptor surface expression [12,13]. Anticancer drugs such as proteasome inhibitors, doxorubicin, cisplatin, histone deacetylase inhibitors (HDACi), topotecan, paclitaxel, etoposide, and tunicamycin increase the expression of death receptors in various tumor cells and sensitize cells to TRAIL [14]. However, the effect of chemotherapeutic agents on the expression of decoy receptors DcR1 and DcR2, which can promote the resistance of the cells to TRAIL, has been poorly investigated.

Modified variants of TRAIL or monoclonal antibodies selective for the death receptor DR4 or DR5 showed increased apoptotic activity in cancer cells and mouse xenograft models [15]. Unfortunately, in clinical trials, TRAIL death receptor agonists did not show a sufficiently high antitumor activity either as separate agents or in combination with various chemotherapeutic drugs [16,17].

Bortezomib (Velcade, PS-341) binds to the β5 subunit of the 26S proteasome, inhibiting its chymotrypsin-like proteolytic activity and preventing the degradation of polyubiquitinated proteins, which leads to cell cycle arrest and activation of apoptosis [18]. Doxorubicin is an anthracycline that acts as a DNA damaging agent, stopping the replication process by stabilizing topoisomerase II, and promotes the formation of free radicals and lipid peroxidation. [19]. Panobinostat (LBH589) is a histone deacetylase (HDAC) inhibitor, which inhibits all enzymes of the HDAC I, II and IV families in vitro and has strong antitumor activity [20]. The potentiation of apoptotic TRAIL signaling by these agents has been demonstrated in vitro and in vivo in various types of tumors [21,22,23,24].

In this study, we examined the effects of chemotherapy such as bortezomib, doxorubicin and panobinostat on the surface expression of TRAIL death and decoy receptors and TRAIL-mediated cell death. Doxorubicin and bortezomib significantly increased surface expression of the death receptors DR4 and DR5, while panobinostat (LBH589) increases only the expression of DR5, but inhibits the expression of DR4. All of these chemotherapeutic drugs also increased the surface expression of DcR1 and DcR2 decoy receptors. The combination of chemotherapy with TRAIL or with the DR5-selective mutant variant DR5-B with no affinity to decoy receptors [25] dramatically reduced the viability of TRAIL resistant A549 and HT-29 cells. However, in sequential exposure, when the cells were pretreated with chemotherapy for 24 h, the TRAIL cytotoxic activity was less pronounced, while DR5-B killed cells quite efficiently. Agonistic antibodies to DR5 have a similar effect. It can be assumed that pretreatment with chemotherapeutic agents can change the ratio of death and decoy receptor surface expression, limiting TRAIL cytotoxic activity.

## 2. Results

### 2.1. Pretreatment of TRAIL-Resistant Cancer Cells with Chemotherapeutic Agents Sensitize Them for DR5-B but Not for TRAIL, While Co-Treatment Was Effective for Both Ligands

Chemotherapeutic agents of various mechanisms of action, such as bortezomib, doxorubicin and panobinostat were selected to sensitize TRAIL-resistant lung adenocarcinoma cells A549 and HT-29 colorectal adenocarcinoma to TRAIL or the DR5 selective TRAIL variant DR5-B.

**Figure 1:**
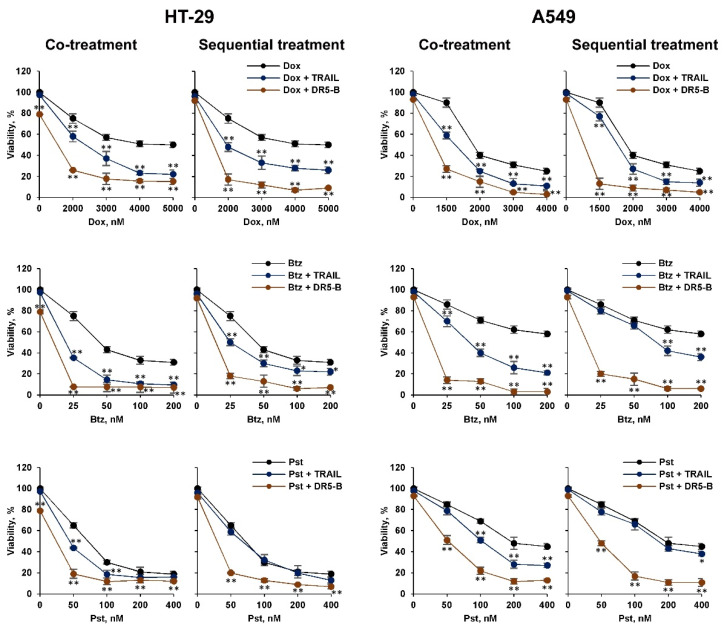
Sensitization of the resistant cancer cells to TRAIL variants by chemotherapeutic agents. In co-treatment experiments, HT-29 and A549 cells were incubated with 1000 ng/mL TRAIL or DR5-B and doxorubicin (Dox), bortezomib (Btz) and panobinostat (Pst) at the indicated concentrations for 48 h. In a sequential treatment, cells were pretreated with chemotherapy for 24 h followed by treatment with TRAIL or DR5-B for another 24 h. Cell viability was determined by WST-1 colorimetric assay. Mean ± Standard Deviation (*n* = 4). The asterisks indicate significance (* *p* < 0.05) and (** *p* < 0.001) relative to cells treated with chemotherapy without ligands. TRAIL—tumor necrosis factor related apoptosis-inducing ligand.

We compared the effects of bortezomib, doxorubicin and panobinostat chemotherapeutic agents on TRAIL and its DR5-selective variant DR5-B-mediated cell death. For this reason, the resistant cancer cells HT-29 and A549 were treated with the chemotherapeutic agents in combination with TRAIL variants either simultaneously or sequentially with chemotherapy followed by ligands. Chemotherapy concentrations were selected based on dose and time-dependent inhibition of cell viability (Figure S1). All three agents sensitized cells to TRAIL and DR5-B in co-treatment experiments, when cells were incubated for 24 h or 48 h, and DR5-B demonstrated higher cytotoxic activity than TRAIL (Figure S2, Figure 1). The profile of the viability curves remained practically unchanged in the co-treated cells after 24 h and 48 h of incubation, except that the absolute cell death was higher when the cells were co-treated with chemotherapy and ligands for 48 h. The difference between the effects of TRAIL and DR5-B was more pronounced when the cells were pretreated with chemotherapy. Moreover, TRAIL was quite ineffective after pretreatment of cells with panobinostat in both cell lines, while DR5-5 efficiency was increased. We hypothesized that chemotherapeutic agents can modulate the expression of both death and decoy receptors, which limits the cytotoxic activity of TRAIL, but not DR5-B.

### 2.2. The Modulation of Surface Expression of TRAIL Receptors and Decoy Receptors by Chemotherapeutic Agents Determines the Effectiveness of Sensitization of Cancer Cells to Ligands

**Figure 2:**
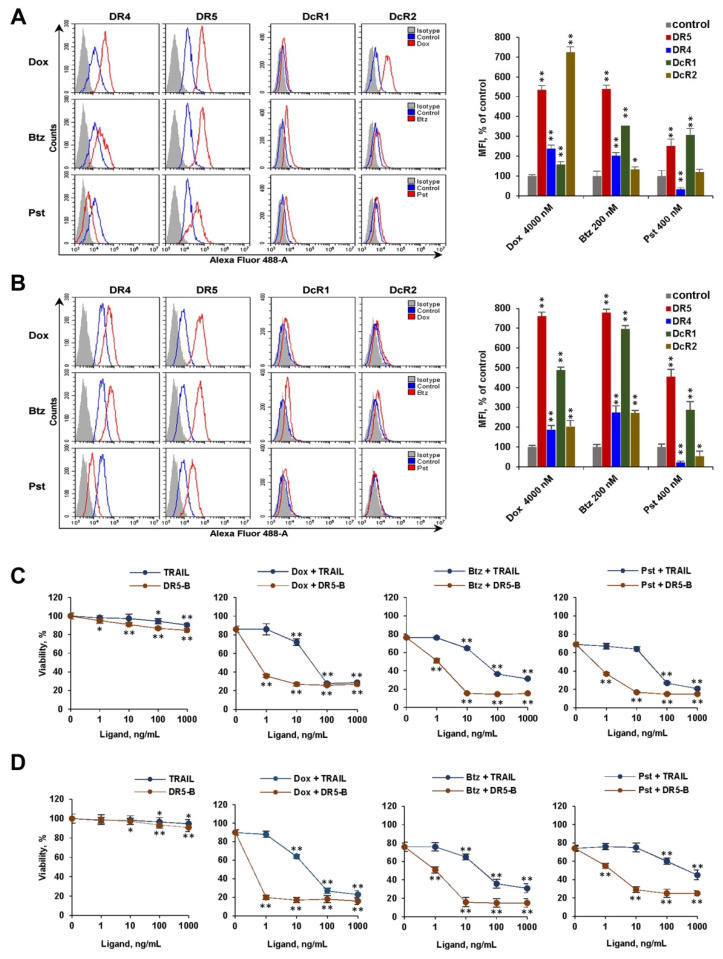
Effect of modulation of surface expression of death and decoy receptors by chemotherapeutic agents on cancer cell sensitization to TRAIL and DR5-B. Surface expression of death and decoy receptors in HT-29 (**A**) and A549 (**B**) cells before and after treatment with the chemotherapeutic agents was determined by flow cytometry. Values of Mean Fluorescence Intensity (MFI) are presented as percent relative to control cells. Representative histograms from three independent experiments with similar results are shown. HT-29 (**C**) and A549 (**D**) cells were co-treated with doxorubicin (4000 nM), bortezomib (200 nM) or panobinostat (400 nM) and TRAIL or DR5-B for 24 h. Cell viability was determined by WST-1 colorimetric assay. Mean ± Standard Deviation (*n* = 3). The asterisks indicate significance (* *p* < 0.05) and (** *p* < 0.001) relative to control cells (**A**,**B**) or relative to cells treated with chemotherapy without ligands (**C**,**D**).

Next, we evaluated the effect of bortezomib, doxorubicin and panobinostat on the surface expression of the TRAIL death and decoy receptors in HT-29 and A549 cells by flow cytometry (Figure 2A,B). Treatment of cells with these agents for 24 h strongly enhanced DR5 expression (5–7 fold) in both cell lines. Bortezomib and doxorubicin also caused an increase in the DR4 receptor (2–2.5 times), while treatment with panobinostat reduced the amount of this receptor on the cell surface in both lines. Chemotherapeutic agents enhanced the surface expression of DcR1 and DcR2 decoy receptors to varying degrees depending on the type of cells, except that panobinostat slightly reduced the expression of DcR2 in A549 cells.

We then compared the efficiency of TRAIL or DR5-B cytotoxicity in combination with chemotherapeutic agents. In both cell lines, DR5-B was highly effective at concentrations of 1–10 ng/mL, while TRAIL killed the cells at concentrations one to two orders of magnitude higher depending on the type of chemotherapy (Figure 2C,D). The affinity of DR5-B to DR5 is not different from TRAIL, as previously demonstrated [18]. Therefore, it can be assumed that the large difference between the effectiveness of TRAIL and DR5-B is due to the expression of decoy receptors DcR1 and DcR2 on the cell surface.

### 2.3. DR5-B Induces Internalization of the DR5 Receptor More Efficiently Than TRAIL

**Figure 3:**
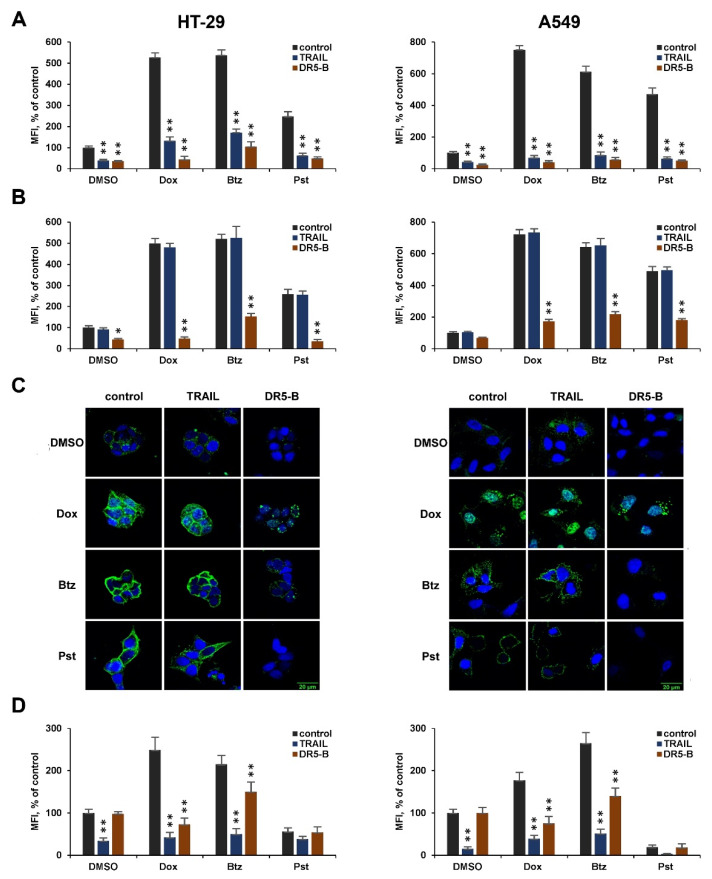
DR5-B causes internalization of DR5 more efficiently than TRAIL. Cells were treated with doxorubicin (4000 nM), bortezomib (200 nM) or panobinostat (400 nM) or with appropriate volumes of dimethyl sulfoxide (DMSO) as a control for 24 h, followed by incubation with TRAIL or DR5-B at a concentration of 1000 ng/mL (**A**) or 10 ng/mL (**B**) for 1 h. Surface expression of DR5 was determined by flow cytometry. Representative histograms from three independent experiments with similar results are shown. (**C**) Cells seeded on a glass slide were treated with chemotherapeutic agents for 24 h, followed by incubation with 10 ng/mL ligands for 1 h. Immunofluorescence staining of the DR5 receptor was analyzed by confocal LSM. (**D**) Surface expression of the DR4 receptor was determined by flow cytometry after treatment of cells with chemotherapy for 24 h, followed by incubation of 1000 ng/mL of ligands for 1 h. Mean ± Standard Deviation (*n* = 3). The asterisks indicate significance (* *p* < 0.05) and (** *p* < 0.001) relative to cells treated with chemotherapy without ligands.

To analyze in more detail the difference in the effects of TRAIL and DR5-B in combination with chemotherapeutic agents, we examined ligand-induced internalization of DR4 and DR5. For this, A549 and HT-29 cells were incubated with chemotherapeutic agents for 24 h, then with ligands for 1 h, and surface expression of receptors was measured by flow cytometry. At a higher concentration (1000 ng/mL), both ligands induced DR5 internalization at almost the same level (Figure 3A). After pretreatment of the cells with chemotherapy, a strong internalization of the DR5 receptor was observed with DR5-B, but not with TRAIL at a concentration of 10 ng/mL (Figure 3B,C). These data indicate that, at low concentrations, TRAIL is titrated by other receptors that limit the activation of DR5-mediated apoptotic signaling. It should be noted that TRAIL and DR5-B caused the internalization of DR5 in TRAIL-resistant cells even without chemotherapeutic agents. However, chemotherapy greatly increased the number of internalized receptors, indicating an improvement in the formation of “death inducing signaling complexes” (DISC), which are responsible for the initiation of apoptotic signaling [26].

TRAIL effectively internalized DR4 either with pretreatment of cells with chemotherapeutic agents, or without it. DR5-B, which does not bind to DR4, did not induce internalization of this receptor in non-pretreated cells. However, after treatment of cells with chemotherapeutic agents, internalization of DR4 under the action of this ligand was observed (Figure 3D). It is possible that increased expression of death receptors on the cell surface promotes the formation of heterodimers where the receptors can be internalized together as part of the same DISC. This assumption is consistent with the fact that death receptors are capable of dimerization due to the interaction of N-terminal domains in the absence of ligands [26,27].

### 2.4. Chemotherapeutic Agents at Low Concentration Effectively Sensitize Cancer Cells to DR5 Specific Agonists, but Not to TRAIL

**Figure 4:**
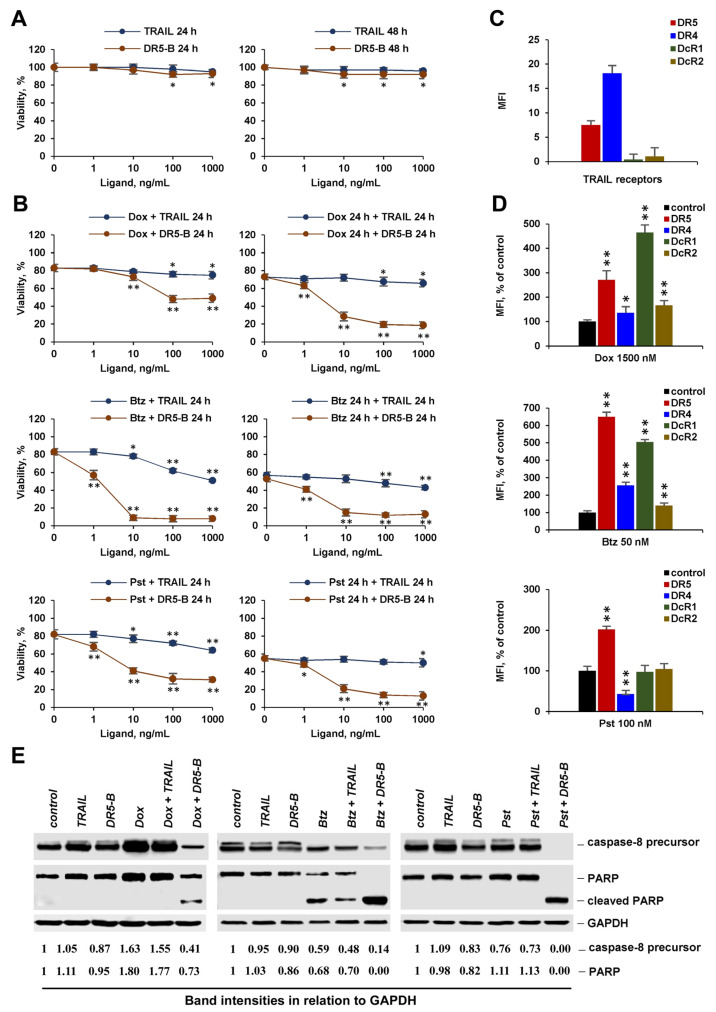
Chemotherapeutic agents at low concentration sensitize A549 cells to DR5-B but not to TRAIL. (**A**) Cells were treated with TRAIL or DR5-B for 24 h and 48 h. (**B**) Cells were treated with TRAIL or DR5-B in combination with 1500 nM doxorubicin (Dox), 50 nM bortezomib (Btz) and 100 nM panobinostat (Pst) for 24 h. In a sequential treatment mode, cells were pretreated with the chemotherapeutic agents for 24 h, followed by treatment with the ligands for another 24 h. Cell viability was quantified by Wst-1 assay. Surface expression of death and decoy receptors before (**C**) and after (**D**) treatment with the chemotherapeutic agents at indicated concentrations determined by flow cytometry. Mean ± Standard Deviation (*n* = 3). The asterisks indicate significance (* *p* < 0.05) and (** *p* < 0.001) relative to cells treated with chemotherapy without ligands. (**E**) Cells were pre-incubated with the chemotherapeutic agents for 16 h, followed by 1000 ng/mL ligands for another 6 h for Dox and Pst and 3 h for Btz. Cleavage of caspase-8 and PARP (Poly(ADP-ribose) Polymerase-1) proteins was analyzed by Western blotting. The intensity of protein bands was calculated using the ImageJ software (http://rsbweb.nih.gov/ij/, NIH, Bethesda, MD, USA) and data were normalized to GAPDH (glyceraldehyde-3-phosphate dehydrogenase).

**Figure 5:**
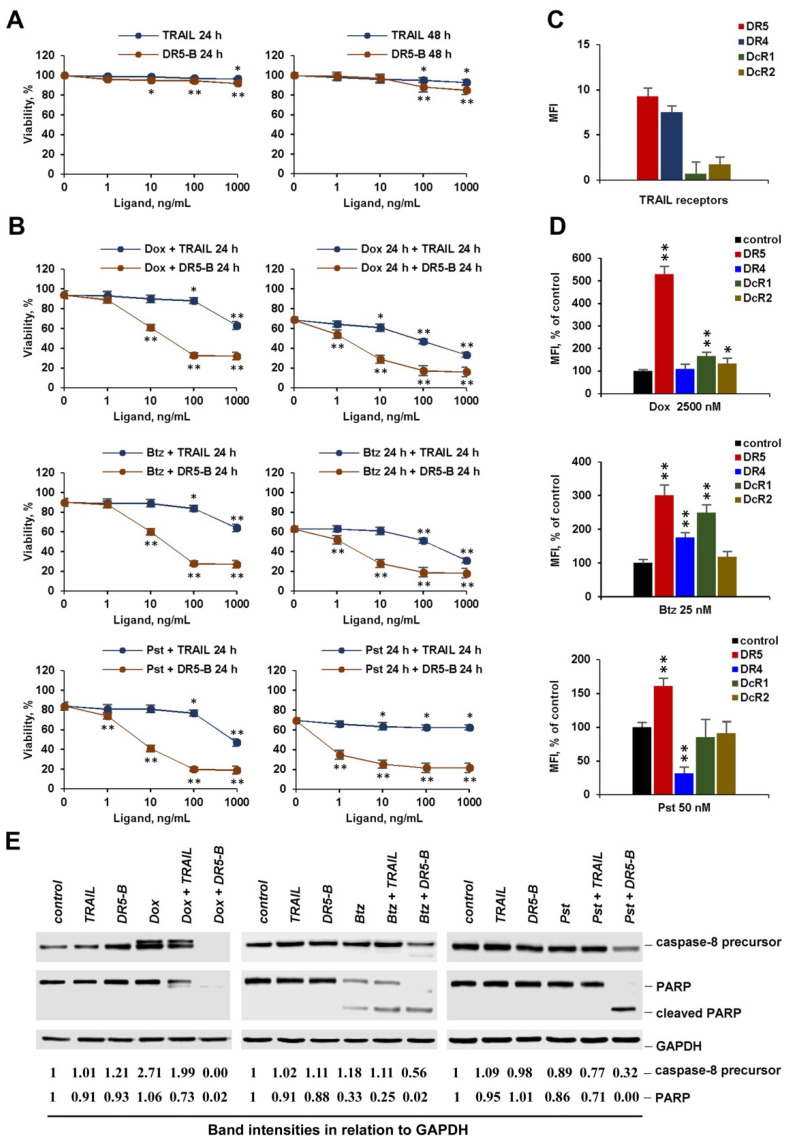
Chemotherapeutic agents at low concentration sensitize HT-29 cells to DR5-B more effectively than TRAIL. (**A**) Cells were treated with TRAIL or DR5-B for 24 h and 48 h. (**B**) Cells were either treated with TRAIL or DR5-B in combination with 2500 nM doxorubicin (Dox), 25 nM bortezomib (Btz) and 50 nM panobinostat (Pst) for 24 h or pretreated with chemotherapy for 24 h, by addition of TRAIL or DR5-B for another 24 h. Cell viability was quantified by Wst-1 assay. Surface expression of death and decoy receptors before (**C**) and after treatment (**D**) with chemotherapy at indicated concentrations determined by flow cytometry. Mean ± Standard Deviation (*n* = 3). The asterisks indicate significance (* *p* < 0.05) and (** *p* < 0.001) relative to cells treated with chemotherapy without ligands. (**E**) Cells were pre-incubated with chemotherapy for 16 h, followed by 1000 ng/mL ligands for another 6 h for Dox and Pst and 3 h for Btz. Cleavage of caspase-8 and PARP proteins was analyzed by Western blotting. The intensity of protein bands was calculated using the ImageJ software (http://rsbweb.nih.gov/ij/, NIH, Bethesda, MD, USA) and data were normalized to GAPDH.

One of the characteristics that distinguish chemotherapeutic agents from other drugs is the frequency and severity of side effects in therapeutic doses. Therefore, reducing the dose of a chemotherapeutic drug in combination therapy can be a promising approach to reduce side effects. For this reason, we studied the effects of doxorubicin, bortezomib, and panobinostat in low concentrations on the cytotoxic activity of TRAIL or death receptor specific agonists in co- and sequential treatment modes. Since the cytotoxic effect of chemotherapy at low concentrations did not significantly differ after 24 h and 48 h, we chose a regimen in which the exposure time of ligands for the combined and sequential treatment was the same (24 h). TRAIL or DR5-B had virtually no effect on the viability of HT-29 and A549 cells after 48 h of incubation (Figure 4A and Figure 5A), despite the expression of death receptors DR4 and DR5 on the cell surface (Figure 4C and Figure 5C). All chemotherapeutic agents effectively sensitized A549 to DR5-B in both modes, while TRAIL was ineffective (Figure 4B). Pretreatment of HT-29 cells with bortezomib and doxorubicin weakly sensitized these cells to TRAIL (Figure 5B). Interestingly, HT-29 cells became completely resistant to TRAIL after pretreatment with panobinostat, while in co-treatment mode, viability was decreased, at least with a high (1000 ng/mL) concentration of TRAIL. The effectiveness of DR5-B in cells pretreated with chemotherapeutic agents was confirmed by cleavage of caspase-8 and PARP proteins detected by Western blotting (Figure 4E and Figure 5E, Figure S3). The reason for TRAIL ineffectiveness may be due to simultaneous stimulation of death and decoy receptor expression by chemotherapeutic agents. For example, a significant increase in the expression of the DcR1 receptor was found at low concentrations of bortezomib and doxorubicin (Figure 4D and Figure 5D). Panobinostat at low concentration increased DR5 expression, while it did not significantly affect decoy receptor expression, but cells were not sensitized to TRAIL. One possible explanation may be related to the participation in the induction of apoptosis of DR4, which was significantly reduced by panobinostat in both cell lines.

**Figure 6:**
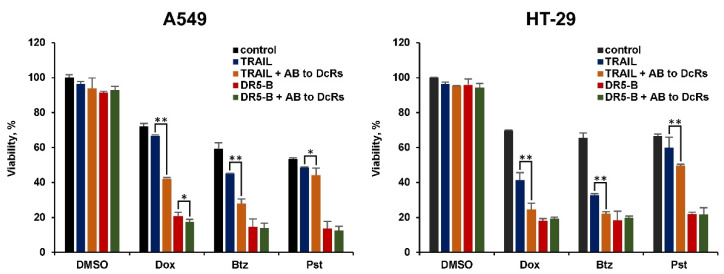
Neutralization of decoy receptors improved TRAIL cytotoxic activity. A549 and HT-29 cells were pre-incubated with 1500 nM and 2500 nM doxorubicin (Dox), 50 nM and 25 nM bortezomib (Btz), 100 nM and 50 nM panobinostat (Pst) respectively for 24 h, following incubation with anti-DcR1 and anti-DcR2 antibodies (15 µg/mL each) for 1 h and 1000 ng/mL ligands for another 24 h. Mean ± Standard Deviation (*n* = 3). The asterisks indicate significance (* *p* < 0.05) and (** *p* < 0.001) relative to cells treated with ligands without anti-DcRs antibodies.

To confirm the role of decoy receptors in the inefficiency of TRAIL, we performed an experiment with antibodies to decoy receptors. After treating the cells with chemotherapy at low concentrations, the cells were incubated for 1 h with antibodies to DcR1 and DcR2 before TRAIL or DR5-B supplementation. Neutralization of decoy receptors significantly improved the cytotoxic activity of TRAIL, especially in cells pretreated with doxorubicin and bortezomib, when the enhancement of decoy receptor expression was more pronounced (Figure 6). As expected, the addition of anti-DcR antibodies did not affect the activity of DR5-B.

**Figure 7:**
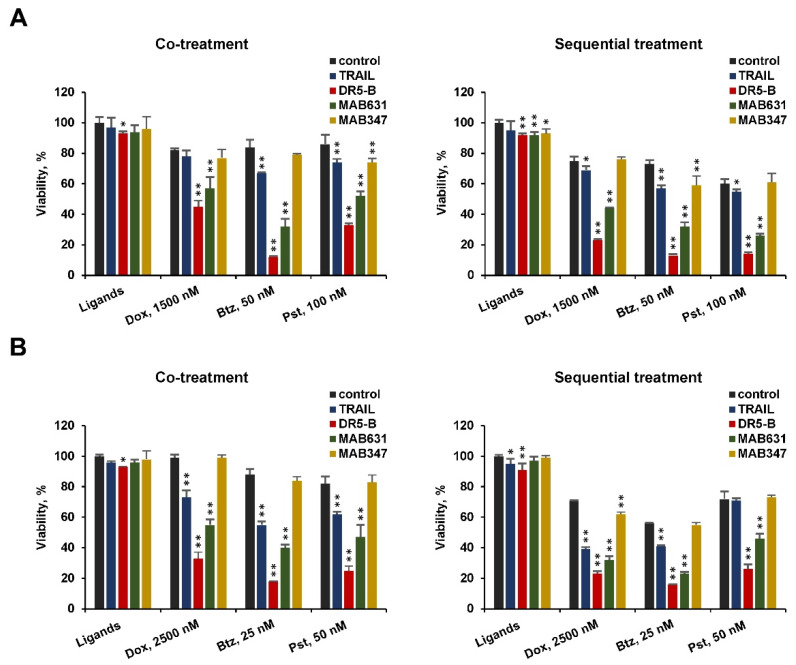
Chemotherapeutic agents sensitize TRAIL-resistant cancer cells to TRAIL mainly by activating DR5 mediated signaling. In co-treatment experiments, A549 (**A**) and HT-29 (**B**) cells were incubated with 1000 ng/mL of TRAIL, DR5-B or with 1000 ng/mL of agonistic antibodies to DR5 (MAB631) or to DR4 (MAB347) in combination with doxorubicin (Dox), bortezomib (Btz) and panobinostat (Pst) at the indicated concentrations for 24 h. In a sequential treatment mode, cells were pretreated with the chemotherapeutic agents for 24 h, followed by treatment with the ligands for another 24 h. Cell viability was quantified by Wst-1 assay. Mean ± Standard Deviation (*n* = 4). The asterisks indicate significance (* *p* < 0.05) and (** *p* < 0.001) relative to control cells or cells treated with chemotherapy without ligands.

Chemotherapeutic agents did not sensitize cells to agonistic DR4 specific antibodies, despite the fact that doxorubicin and bortezomib enhanced the expression of this receptor (Figure 7). In contrast, DR5 specific antibodies effectively reduced the viability of A549 and HT-29 cells both in co-treated or sequential treated cells, indicating that chemotherapy mainly activated DR5 signaling. It should be noted that both anti-DR4 and anti-DR5 antibodies effectively reduced the viability of HCT116 cells, thereby confirming the functional activity of the antibodies used in this study (data not shown). Blocking the expression of DR5 but not expression of DR4 using small interfering RNA has been shown to make cells less susceptible to apoptosis caused by a combination of bortezomib with TRAIL in human Non-small-cell lung carcinoma (NSCLC) cells [28].

## 3. Discussion

The natural and acquired resistance of tumors to TRAIL and monoclonal antibodies to death receptors DR4 or DR5 does not yet allow success in using these drugs in clinical practice, despite the high antitumor activity of these preparations in animal models [17]. The mechanisms involved in the development of resistance to TRAIL in many cancer cells are still not entirely understood. The combination of DR agonists with numerous traditional anticancer drugs is a common strategy to overcome the resistance of tumors to TRAIL [29]. These agents usually decreased the expression of anti-apoptotic (often c-FLIP, NF-kB, XIAP, BCL-2) and enhanced the expression of pro-apoptotic proteins (DR4, DR5, FADD, CHOP, Bax, Bad) [30]. Enhanced expression of death receptors by numerous natural and chemical agents overcame the resistance of cancer cells to TRAIL [13,15]. Upregulation of death receptors by doxorubicin [24,31], bortezomib [28,32,33] and panobinostat [34] was observed in different types of cancer cells. However, the effect of anticancer drugs on the expression of the decoy receptors DcR1 and DcR2 was poorly studied, although the inhibitory potential of decoy receptors for antitumor activity of TRAIL has been established in many studies [35,36,37,38,39]. Upregulation of DcR1 expression was observed by oxaliplatin in wild-type p53 colon cancer cells, which limited the synergistic antitumor potential induced by TRAIL [40].

Here we have demonstrated that chemotherapeutic agents such as doxorubicin, bortezomib and panobinostat simultaneously stimulate the expression of TRAIL death and decoy receptors in cancer cells. Despite this, they sensitized TRAIL-resistant cancer cells to TRAIL at high ligand concentrations. At the same time, the DR5-selective variant of DR5-B, which does not bind to decoy receptors DcR1 and DcR2, in combination with the chemotherapeutic agents killed cells at 10–100 times lower concentrations compared to TRAIL. For example, in combination with doxorubicin, DR5-B was able to kill HT-29 and A549 cells at a concentration of 1 ng/mL, while for the same inhibition of cell viability, 100 times more TRAIL was required. We suggested that titration by binding to decoy receptors is the main reason for the low effectiveness of TRAIL, as confirmed by studies on the internalization of DR5 and DR4 receptors. At a higher concentration (1000 ng/mL), both ligands induced the internalization of DR5 at the same level before or after treatment of cells with chemotherapy. However, at a concentration of 10 ng/mL, DR5-B induced effective internalization of DR5, while TRAIL was completely ineffective, most probably due to titration by binding to decoy receptors. The DR4 receptor was not internalized by DR5-B in non treated cells. Interestingly, partial internalization of DR4 was induced by DR5-B after treatment of the cells with doxorubicin or bortezomib, which suggests the formation of heterodimers of death receptors after increasing their surface expression under the influence of chemotherapeutic agents [41].

Even if chemotherapeutic agents enhance the surface expression of both death receptors, cell sensitization occurs by activating signal transmission mainly through DR5, since agonistic antibodies to DR4 practically do not cause cell death. In contrast, cell killing with specific anti-DR5 antibodies was more effective than with TRAIL. We previously showed that the combination of TRAIL and bortezomib leads to the internalization and degradation of DR4 in HCT116 cells, regardless of p53 status [42].

Finally, very low doses of chemotherapeutic agents sensitized TRAIL-resistant cells to DR5-B, when TRAIL effectiveness was restricted. It was shown earlier that doxorubicin at low concentrations sensitizes human solid cancer cells to the DR5 specific antibody lexatumumab by upregulation of DR5 expression [43]. Doxorubicin and bortezomib at low concentrations still stimulated surface expression of DcR1, which can acts as a competitor for TRAIL, preventing DR5-associated DISC assembly [4]. We demonstrated that panobinostat at low concentrations does not affect the expression of decoy receptors, but reduces the amount of DR4. The ineffectiveness of TRAIL in combination with panobinostat can be explained by weaker stimulation of DR5 expression at low drug concentrations.

The effect of chemotherapeutic agents on cytotoxic activity of DR agonists in cancer cell lines was mainly studied in co-treatment mode. It was shown that simultaneous treatment with TRAIL and etoposide fails to cooperate to induce apoptosis in the colon cancer cell line HCT116 Bax^−/−^, and pretreatment of cells with etoposide for 48 h followed by TRAIL overcomes Bax deficiency [44]. However, when Bax deficiency was associated with ectopic expression of DcR2, this combination did not restore TRAIL-induced apoptosis. Here we have demonstrated that pretreatment of cancer cells with chemotherapy can limit the cytotoxic activity of TRAIL by enhancing the expression of decoy receptors. Thus, DR5-specific agonists are preferable in combination regimens with chemotherapy as they are able to sensitize cancer cells at low concentrations of chemotherapy independently of administration mode. In addition, combination therapy with lower doses of chemotherapy is safer because chemotherapeutic agents exhibit various side effects at higher doses. Common side effects at higher doses of doxorubicin, bortezomib and panobinostat are cardiotoxicity, thrombocytopenia and neuropathy respectively [45,46,47].

## 4. Materials and Methods

### 4.1. Cell Culture and Reagents

Human lung adenocarcinoma cell line A549 and human colorectal adenocarcinoma cell line HT-29 were acquired at the Scientific Research Institute of Cytology, Russian Academy of Sciences (St. Petersburg, Russia). Nutrient media for cell culture DMEM and RPMI 1640, 0.05% trypsin solution with EDTA, and phosphate-buffered saline were from PanEco (Moscow, Russia), bovine fetal serum was from HyClone (Cramlington, UK), doxorubicin was from Tocris (Bristol, UK), and panobinostat and bortezomib were from Santa Cruz (Dallas, TX, USA). Antibodies to decoy receptors DcR1 (AF630) and to DcR2 (AF633) were form R&D systems.

### 4.2. Expression and Purification of Recombinant Preparations TRAIL and DR5-B

Recombinant proteins of the wild-type extracellular domain TRAIL (114–280) and its DR5-specific variant DR5-B were expressed in *Escherichia coli* and purified as previously described [48]. The *E. coli* SHuffle B strain was transformed by pET32a/sdr5-b or pET32a/strail plasmids and cell cultures were grown at 28 °C for 20 h. After disruption of the cells by French Press (Spectronic Instruments Inc., Rochester, NY, USA), TRAIL and DR5-B were purified from the cytoplasmic protein fraction by immobilized metal-affinity chromatography on Ni-NTA agarose (Qiagen, Germantown, MD, USA). Final purification was carried out on SP Sepharose column (GE Healthcare, Danderyd, Sweden) and the endotoxins were eliminated using Pierce High Capacity Endotoxin Removal Resin (Thermo Fisher Scientific, Waltham, MA, USA). Purified protein solutions were dialyzed against 150 mM NaCl for 24 h at 4 °C, sterilized by filtration, lyophilized and stored at −70 °C.

### 4.3. Cell Viability Test

A549 cells were cultured in DMEM nutrient medium supplemented with 10% fetal bovine serum. HT-29 cells were cultured in RPMI 1640 medium supplemented with 10% fetal bovine serum. Cells were seeded in 96-well plates at a density of 1 × 10^4^ per well in 100 μL culture medium and incubated for 24 h in humidified atmosphere of 5% CO_2_ in air (New Brunswick, Eppendorf, Germany) at 37 °C. Culture medium was aspirated and 100 μL of fresh serum free medium supplemented with TRAIL variants and chemotherapeutic drugs was added to wells. After 24 h incubation, 10 µl WST-1 reagent (Sigma-Aldrich, St. Louis, MO, USA) was added to each well and the plates were kept for 2 h at 37 °C. The optical density of the wells was measured using an iMark plate spectrophotometer (Bio-Rad, Hercules, CA, USA) at a wavelength of 450 nm with background subtraction at 655 nm.

### 4.4. Flow Cytometry

The cells were seeded in 6 well plates at a density of 2 × 10^5^ cells per well in 2 mL of culture media. After 24 h incubation, the cells were washed with serum free medium and incubated with chemotherapeutic agents for 24 h. After TRAIL or DR5-B was added to the wells to the final concentration of 1000 ng/mL and incubated for 1 h. Cells were detached from culture flasks using 2 mM EDTA solution, washed in ice cold PBS, and resuspended in FACS buffer (PBS with 1% BSA). Monoclonal antibodies to TRAIL receptors DR4 (DR-4-02), DR5 (DR5-01-1) (GeneTex, Irvine, CA, USA), DcR1 (TR3.06) and DcR2 (TRAIL-R4-01) (Enzo Life Sciences, Farmingdale, NY, USA) were added to the cell suspension to a final concentration of 5 μg/mL and incubated for 1 h at 4 °C. Then the cells were washed twice with cold FACS buffer, and incubated with 10 μg/mL secondary antibodies AlexaFluor 488 (Invitrogen, Waltham, MA, USA) for 1 h at 4 °C, washed twice with cold FACS buffer, and suspended in FACS buffer supplemented with 1 μg/mL of propidium iodide. Mouse IgG1 (15H6, GeneTex, Irvine, CA, USA) was used as isotype control. Samples were analyzed on a CytoFlex flow cytometer (Beckman Coulter, Indianapolis, IN, USA).

### 4.5. Western Blotting

For each sample, 1.5 × 10^6^ cells were seeded on 100 mm cell culture dishes and after treatment with chemotherapeutic agents or TRAIL and DR5-B, flasks were washed once with PBS, and cells were detached by 2 mM EDTA solution. Cells were harvested by centrifugation at 500× *g* for 5 min and suspended in 0.3 mL RIPA buffer (Sigma-Aldrich), incubated for 30 min on ice and subsequently centrifuged for 15 min at 16,000× *g*. The protein concentration in supernatants was measured using a Micro BSA Protein Assay Kit (Thermo Fisher Scientific) according to the manufacturer’s instructions. Samples containing 20 μg protein were heated at 95 °C for 5 min, subjected to reducing sodium dodecyl sulfate polyacrylamide gel electrophoresis (SDS-PAGE) and transferred onto polyvinylidene difluoride membranes (Life Technologies, Eugene, OR, USA). The membrane was blocked in TBST (50 mM Tris-HCl, 150 mM NaCl with 0.05% of Tween-20) containing 5% nonfat dry milk for 2 h. After washing with TBST three times for 10 min, the membrane was incubated with the primary antibodies anti-caspase 8 (5F7) (Enzo Life Sciences, Farmingdale, NY, USA), anti-PARP1 (123) or anti-GAPDH (GA1R) (Thermo Fisher Scientific) in TBST/milk solution for 1 h at room temperature. After three washes in TBST for 5 min each, the blots were incubated with Horseradish Peroxidase conjugated isotype-specific secondary antibody (HAF007, R&D Systems, Minneapolis, MN, USA) diluted 1:1000 in TBST for 1 h. After washing three times for 5 min with TBST, the blots were developed by Clarity™ Western ECL Substrate (Bio-Rad). The blots were visualized on Versa Doc MP4000 documentation system (Bio Rad, Hercules, CA, USA).

### 4.6. Confocal Microscopy

Glass slides were placed in 6 well plates, 2 × 10^5^ cells were seeded in each well and allowed to grow for 24 h. The culture media was replaced with fresh media without FBS supplemented with chemotherapeutic agents, and after 24 h incubation, 10 ng/mL of TRAIL variants were added to the growth medium. After 1 h incubation with ligands at 37 °C, the cells were washed with ice cold PBS and fixed in 3% paraformaldehyde for 20 min. Since the target protein was of a transmembrane nature, the permeabilization step was skipped. After washing with ice-cold PBS and blocking in 3% BSA in PBS for 30 min, primary antibodies to death receptor DR5 DR5-01-1 (GeneTex, Irvine, CA, USA) were added at a concentration of 2 µg/mL diluted in 3% BSA/PBS and the cells were incubated for 1 h at room temperature. The cells were washed three time with PBS and the glass slides were incubated with Alexa Fluor 488 goat anti-mouse IgG (Invitrogen, Waltham, MA, USA) and Hoechst 33342 in the dark for 1 h at room temperature. Confocal LSM analysis was performed on Leica TCS SP (Leica microsystems, Wetzlar, Germany) equipped with immersion ×100 objective with a 1.4 digital aperture.

### 4.7. Statistical Analysis

Cell culture assays were repeated at least three times and the mean value ± standard deviation was calculated. The significance of the differences was determined using the Student’s t-test Microsoft Office Excel 2013 (Redmond, WA, USA) software.

## 5. Conclusions

Anticancer drugs can simultaneously upregulate death and decoy receptor surface expression, which may limit the antitumor activity of TRAIL in combination regimens. DR5-selective agonists such as DR5-B or monoclonal antibodies to DR are preferred for sensitization of resistant cancer cells and most likely tumors in combination therapy regimens.

## Supplementary Materials

The following are available online at https://www.mdpi.com/2072-6694/12/5/1129/s1, Figure S1: Dose and time-dependent inhibition of cell viability by chemotherapeutic drugs. Figure S2: Sensitization of the resistant cancer cells to TRAIL variants by chemotherapeutic agents. Figure S3: Western blot analysis of caspase-8 and PARP proteins after sequential treatment of cells with chemotherapy and TRAIL or DR5-B.
