# Supplementary material for: Chemotherapeutic Agents Sensitize Resistant Cancer Cells to the DR5-Specific Variant DR5-B More Efficiently Than to TRAIL by Modulating the Surface Expression of Death and Decoy Receptors
Source: Cancers (Basel). 2020 Apr 30;12(5):1129. doi: 10.3390/cancers12051129 (PMC7280987; doi:10.3390/cancers12051129)
Supplement: Supplementary file 1 [file cancers-12-01129-s001.pdf]

cancers

MDPI

Supplementary Materials

Chemotherapeutic Agents Sensitize Resistant Cancer Cells to the DR5-Specific Variant DR5-B more Efficiently than to TRAIL by Modulating the Surface Expression of Death and Decoy Receptors

Artem A. Artykov, Dmitry A. Belov, Victoria O. Shipunova, Daria B.Trushina, Sergey M. Deyev, Dmitry A. Dolgikh, Mikhail P. Kirpichnikov, Marine E. Gasparian

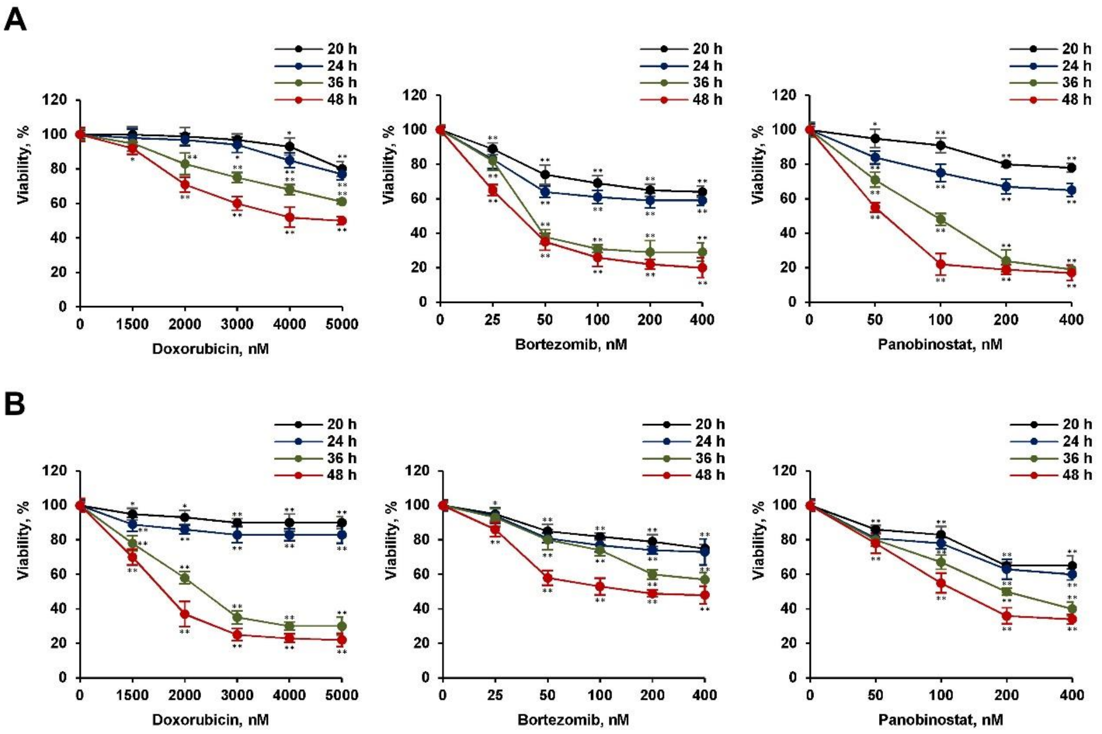

Figure S1. Dose and time-dependent inhibition of cell viability by chemotherapeutic drugs. HT-29 (**A**) and A549 (**B**) cells were incubated with indicated concentrations of doxorubicin (Dox), bortezomib (Btz) and panobinostat (Pst) for 20, 24, 36 and 48 h and Cell viability was determined by WST-1 colorimetric assay. Mean ± Standard Deviation ($n = 4$). The asterisks indicated significance (* $p < 0.05$) and (** $p < 0.001$) relative to control cells.

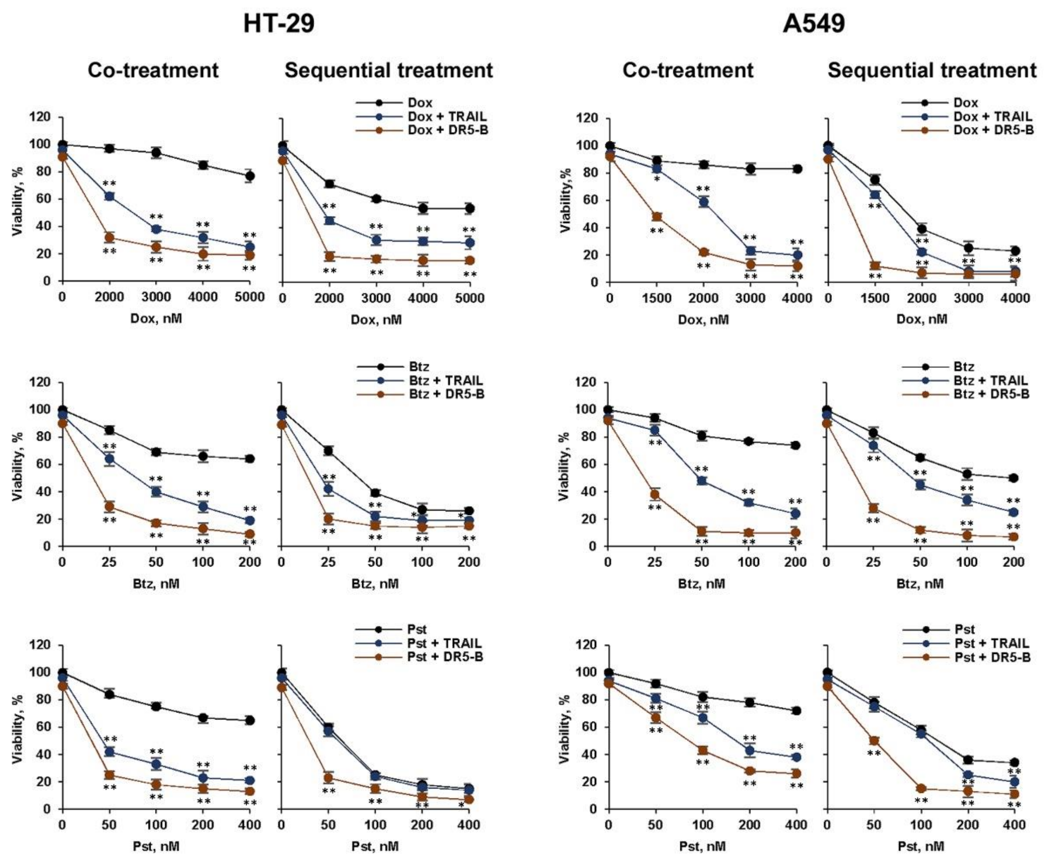

Figure S2. Sensitization of the resistant cancer cells to wild type TRAIL and DR5-selective variant DR5-B by chemotherapeutic agents. In co-treatment experiments HT-29 and A549 cells were incubated with 1000 ng/mL TRAIL or DR5-B and doxorubicin (Dox), bortezomib (Btz) and panobinostat (Pst) at the indicated concentrations for 24 h. In a sequential treatment, cells were pretreated with chemotherapy for 24 h followed by treatment with TRAIL or DR5-B for another 24 h. Cell viability was determined by WST-1 colorimetric assay. Mean ± Standard Deviation (n = 4). The asterisks indicated significance (* $p < 0.05$) and (** $p < 0.001$) relative to cells treated with chemotherapy without ligands.

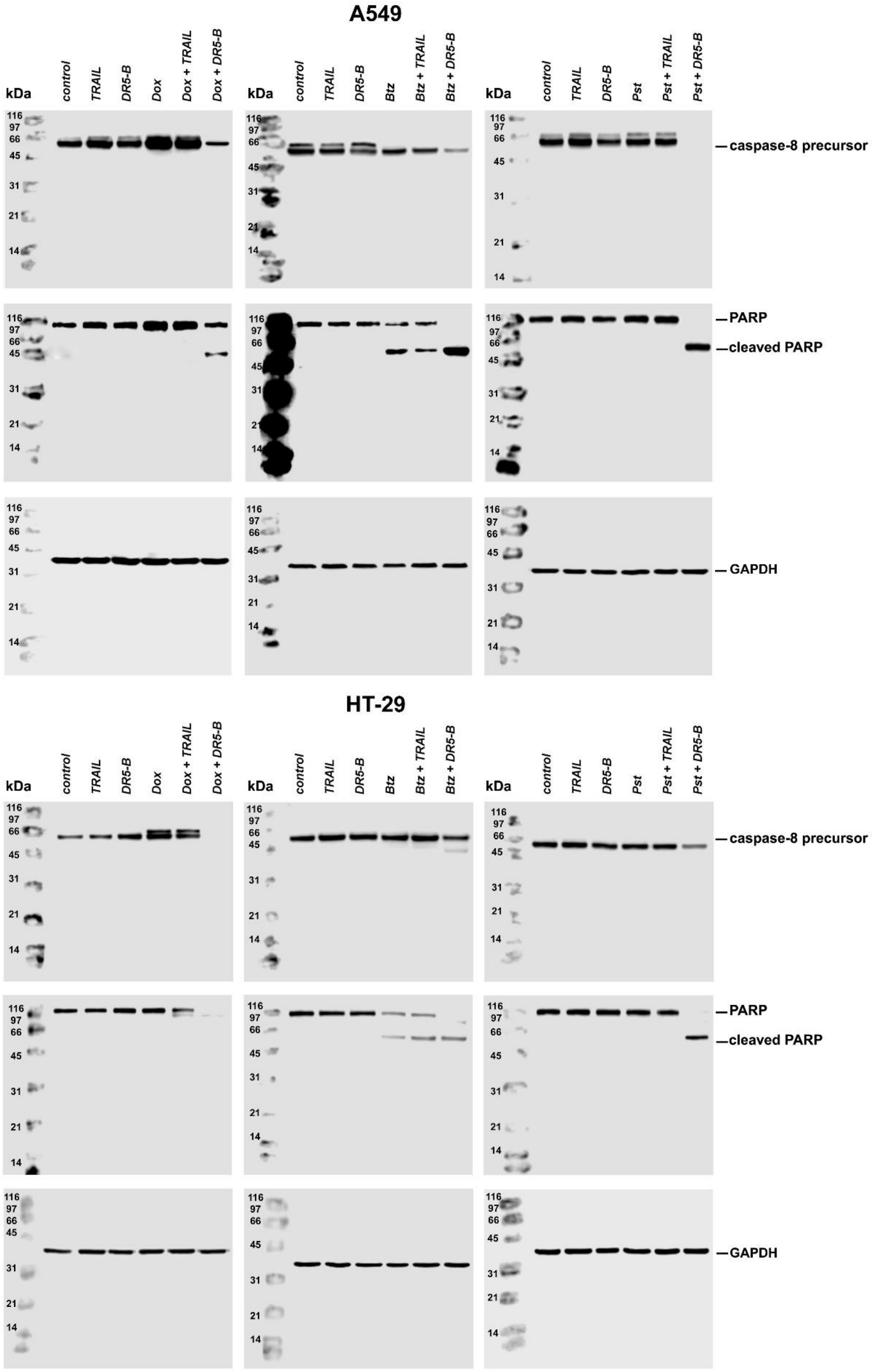

Figure S3. Western blot analysis of caspase-8 and PARP (Poly(ADP-ribose) Polymerase-1) proteins after sequential treatment of cells with chemotherapy and TRAIL or DR5-B. GAPDH (glyceraldehyde-3-phosphate dehydrogenase) was used for normalization of protein bands. Cells were pre-incubated with 1500 nM and 2500 nM doxorubicin (Dox), 50 nM and 25 nM bortezomib (Btz), 100 nM and 50 nM panobinostat (Pst) for A549 and HT-29 respectively for 16 h, followed by

1000 ng/mL ligands for another 6 h for Dox and Pst and 3 h for Btz. Protein concentration was determined using a Micro BSA Protein Assay Kit (Thermo Fisher Scientific) according to the manufacturer's instructions. Samples containing 20 μg protein were heated at 95 °C for 5 min, subjected to reducing sodium dodecyl sulfate polyacrylamide gel electrophoresis and transferred to polyvinylidene difluoride membranes. To mark the position of molecular markers, polyvinylidene difluoride (PVDF) membranes after trans-blotting were stained with 0.1% Ponceau S in 5% acetic acid and marked with Western Blot Marker chemiluminescence Pen.
